# A review on the impact of single-stranded library preparation on plasma cell-free diversity for cancer detection

**DOI:** 10.3389/fonc.2024.1332004

**Published:** 2024-03-06

**Authors:** Jordan C. Cheng, Neeti Swarup, David T. W. Wong, David Chia

**Affiliations:** ^1^ School of Dentistry, University of California, Los Angeles, Los Angeles, CA, United States; ^2^ Stanford Cancer Institute, Stanford University, Stanford, CA, United States; ^3^ Department of Pathology and Laboratory Medicine, David Geffen School of Medicine, University of California, Los Angeles, Los Angeles, CA, United States

**Keywords:** cell-free DNA, liquid biopsy, single-stranded library preparation, fragment-size, ultrashort single-stranded cell-free DNA

## Abstract

In clinical oncology, cell-free DNA (cfDNA) has shown immense potential in its ability to noninvasively detect cancer at various stages and monitor the progression of therapy. Despite the rapid improvements in cfDNA liquid biopsy approaches, achieving the required sensitivity to detect rare tumor-derived cfDNA still remains a challenge. For next-generation sequencing, the perceived presentation of cfDNA is strongly linked to the extraction and library preparation protocols. Conventional double-stranded DNA library preparation (dsDNA-LP) focuses on assessing ~167bp double-stranded mononucleosomal (mncfDNA) and its other oligonucleosomal cell-free DNA counterparts in plasma. However, dsDNA-LP methods fail to include short, single-stranded, or nicked DNA in the final library preparation, biasing the representation of the actual cfDNA populations in plasma. The emergence of single-stranded library preparation (ssDNA-LP) strategies over the past decade has now allowed these other populations of cfDNA to be studied from plasma. With the use of ssDNA-LP, single-stranded, nicked, and ultrashort cfDNA can be comprehensively assessed for its molecular characteristics and clinical potential. In this review, we overview the current literature on applications of ssDNA-LP on plasma cfDNA from a potential cancer liquid biopsy perspective. To this end, we discuss the molecular principles of single-stranded DNA adapter ligation, how library preparation contributes to the understanding of native cfDNA characteristics, and the potential for ssDNA-LP to improve the sensitivity of circulating tumor DNA detection. Additionally, we review the current literature on the newly reported species of plasma ultrashort single-stranded cell-free DNA plasma, which appear biologically distinct from mncfDNA. We conclude with a discussion of future perspectives of ssDNA-LP for liquid biopsy endeavors.

## 1 Introduction

### 1.1 Cell-free DNA

Liquid biopsy, which harnesses biomolecules within biofluids to infer the characteristics and activity of a distant primary tumor of cancer within the body, has emerged from its infancy into a bustling field of study (1). Although tumor-derived cells, proteins, or metabolites spearheaded the initial liquid-biopsy interest, cell-free DNA (cfDNA) has now become the most highly focused analyte. Cell-free DNA is thought to be derived from degraded DNA from a variety of mechanisms (apoptosis, necrosis, or secretion (2)) and is detectable by many technologies, especially next-generation sequencing (NGS). There are various conformations of cfDNA present in blood plasma and serum, saliva, urine, and cerebral spinal fluid with unique characteristics. Therefore, cell-free DNA has become an important part of liquid biopsy workflow. In cancer diagnosis, one potential attribute of cfDNA is the ability to assay for tumor-derived cfDNA, referred to as circulating tumor DNA (ctDNA). Studying ctDNA involves the examination of cfDNA fragments that contain signature mutant signals such as single base pair mutations (3), amplifications (4, 5), fragment-size changes (6), methylation (7), or other discrimination topological features (8).

Despite its many virtues, due to its rarity, the detection of ctDNA is challenging and has been alluded to finding a needle in a haystack. Since non-cancer cells undergo constant replication and controlled death cycles, ctDNA is present at extremely low concentrations compared to cfDNA of non-tumor origin (9). For cfDNA, reported tumor DNA to wildtype sequences ratios range from >5-10% at later stages, which is feasible for detection, to increasingly rare ratios of <0.01 to 0.1% at early stages (or after surgical intervention) (10).

Adding to this complexity, the overall understanding of the biology and size distribution has not been established. The predominant type of cfDNA analyzed by current assays is the double-stranded 167-bp mononucleosomal cell-free DNA (mncfDNA) molecule and those derived from di- or tri- nucleosomes (11). The 167-bp cell-free DNA link to histones has been well established (12–14). The observed cfDNA structure and size can be dependent on the mechanism of release from cells. The appearance of cfDNA can be altered depending on if it is derived from cell necrosis, apoptosis, phagocytosis, and extracellular versicles release (15). During apoptosis, DNA processing creates the iconic pattern of fragments presenting in multiples of 180-200bp. DNA wrapped around histone octomers is 147bp in length, with a linker DNA ranging from 20-90bp. However, the cfDNA population also contains other conformations of DNA, including single-stranded, nicked, and jagged DNA of different sizes. These may not be comprehensively documented in all assays depending on the inherent nature of the analytical strategies.

## 2 cfDNA library preparations

### 2.1 Double-stranded DNA library preparation

**Figure 1:**
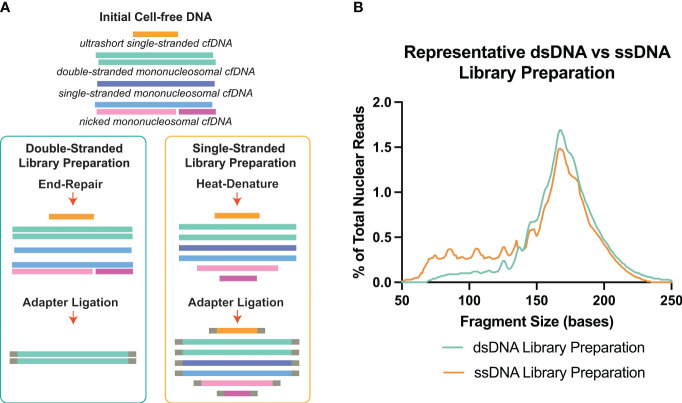
Double-stranded DNA (dsDNA) and single-stranded DNA (ssDNA) library preparation incorporates different DNA species from cell-free DNA. **(A)** Principal differences between the two library preparation methodologies. The initial heat-denature step in single-stranded library preparation allows the inclusion of multiple conformations of cell-free DNA. **(B)** Representative fragment profiles generated by double-stranded and single-stranded library preparations show that single-stranded library preparations are more sensitive for representing shorter cfDNA fragments below 80bp. Data has been derived from (11, 21).

NGS which provides basepair resolution of each incorpable DNA molecule in the sample, has been an effective method to assess the fragment size nature and associated sequence of cell-free DNA molecules. Traditionally, cfDNA analysis has been focused on double-stranded DNA (16–18). Double-stranded library preparation (dsDNA-LP) is accessible and affordable per sample and since its debut, it has been progressively optimized. During adapter ligation, for double-stranded library preparation, the overhangs of each dsDNA molecule must be polished, causing the dsDNA molecule to lose a portion of its original sequence (19, 20) (**Figure 1A**). Another aspect of the dsDNA-LP is that it is unable to incorporate short, degraded single-stranded DNA or those with single-strand breaks (nicks) (19, 22). Therefore, although it is established as a biomolecular tool, it is unable to assess all possible populations within each biological sample.

### 2.2 Single-stranded DNA library preparation

The emergence of ssDNA-LP protocols, initially arose from the need for ancient DNA analysis, which dealt with greatly fragmented and degraded DNA samples within fossilized remains (22, 23). By utilizing a single-stranded library preparation, investigators were able to sequence the genome of a fossilized extracted DNA, which, through time, frequently become fragmented and single-stranded (22, 24). Svante Pääbo, who led these studies, eventually received the Nobel Prize for Physiology or Medicine in 2022 (25).

There are certain considerations when choosing dsDNA-LP vs ssDNA-LP workflows (**Table 1**). Single-stranded DNA library preparations require the heat denaturation of duplex template DNA, separating the molecules into two single-stranded templates prior to adapter ligation (**Figure 1A**). This denaturation allows for the incorporation of both blunt end and nicked dsDNA and ssDNA molecules. Therefore, by default, single-stranded libraries do not exclusively incorporate single-stranded molecules since they convert all DNA molecules to their single-stranded form. Additionally, since no end-repair is performed, the ends remain unaltered, making it possible to explore the native patterns of DNA fragmentation (26). In cell-free DNA, the use of single-stranded libraries has demonstrated an elevation in cfDNA molecules shorter than 100bp (11, 27–29) (**Figure 1B**).

**Table 1:** Comparison between dsDNA-LP and ssDNA-LP workflows.

Considerations	dsDNA-LP	ssDNA-LP
Commercial Kit Availability	Many	Limited
Unique Molecular Identifier Availability	Many	Limited
Provides dsDNA Native Duplex Information	Yes	No
End Repair Required	Yes	No
Informs Native Fragment Length	No	Yes
Informs Native End-Motif Sequences	No	Yes
Recovers Single-stranded DNA	No	Yes
Recovers Jagged-End DNA	No	Yes
Recovers Nicked DNA	No	Yes
Recovers Shorter Molecules	No	Yes

## 3 Single-stranded DNA ligation strategies

**Figure 2:**
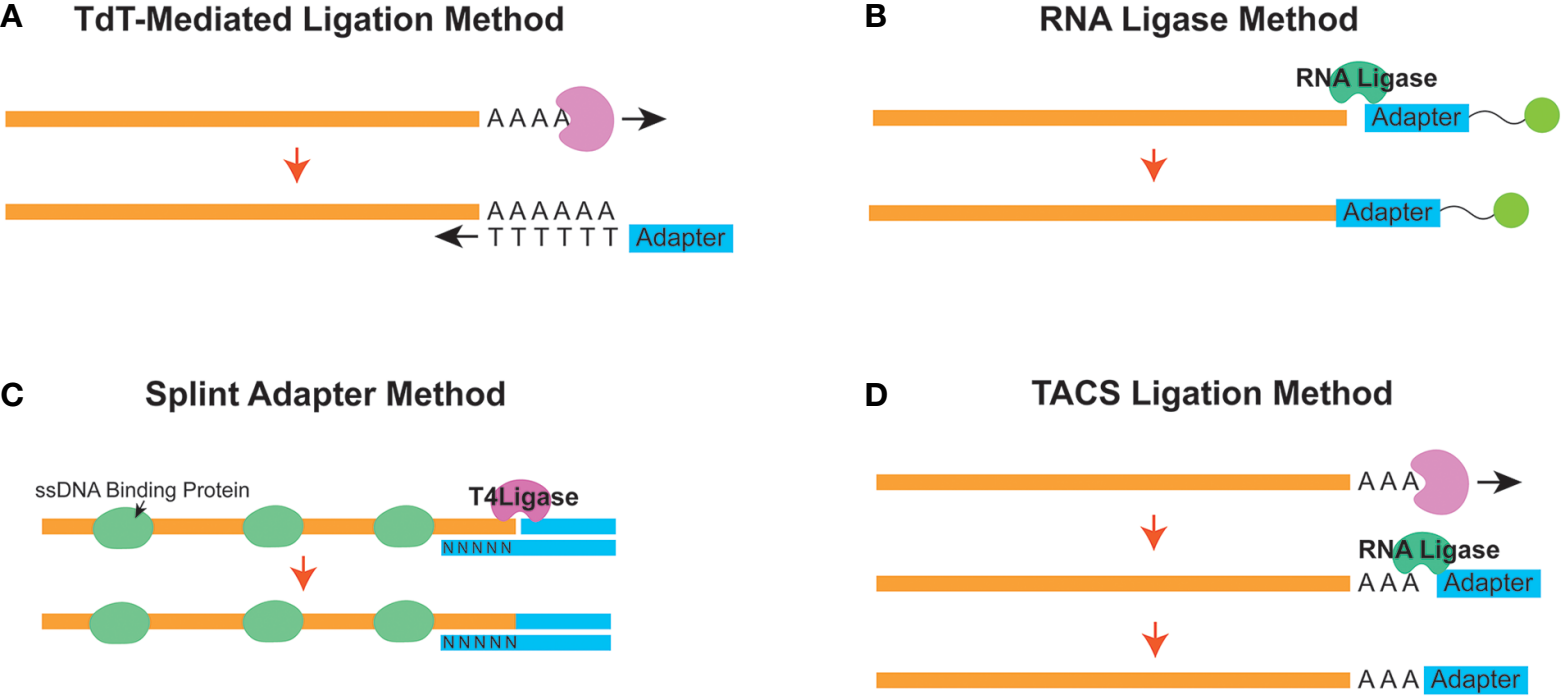
Different strategies to append the initial adapter to a single-stranded molecule in a single-stranded DNA library preparation workflow. Schematic diagrams for the **(A)** Terminal Deoxynucleotidyl Transferase (TDT) - Mediated Ligation, **(B)** RNA Ligase, **(C)** Splint Adapter, and **(D)** TDT-assisted adenylate connector-mediated single-stranded (ss) DNA (TACS) methods are shown.

A common protocol of all ssDNA-LP protocols is a heat-denature step (**Figure 1A**). Subsequently, in order to prepare the library for downstream sequencing, a method is required to attach the sequencing primer sequences to one end of the ssDNA molecule. The ligation strategy is essentially what unlocks the ability to fabricate complete NGS libraries. Many research groups have developed sophisticated methods for ssDNA library preparation, improving on limitations and caveats (**Figure 2**). The following are the current strategies for adapter ligation for single-stranded library preparation:

### 3.1 Terminal deoxynucleotidyl transferase-mediated tailing

Terminal deoxynucleotidyl transferase (TdT)-mediated tailing is a strategy (**Figure 2A**) where the TdT enzyme is used to append a homopolymeric tail of adenosine or thymine nucleotides to the 3’-end of a ssDNA molecule (30). This resulting homopolymeric nucleotide tail can be used as a hybridization priming site for a complementary primer (31). Once the tail hybridizes with the primer, the cfDNA can be converted from a ssDNA molecule into one that is double-stranded. Subsequently, once converted, a sequencing second adapter on the 5’-end can be ligated using T4 DNA ligase (32). However, the homopolymeric tails can cause confusion downstream during downstream analysis since the investigator will need to be able to differentiate between the native and synthetically introduced nucleotides.

### 3.2 RNA ligase-based ligation

Another adapter ligation strategy uses the ability of the RNA ligase enzyme to conjoin a 5’-phosphorylated adapter to the 3’ end of the ssDNA molecule. This strategy was first introduced from ancient DNA workflows and has been implemented to assess cfDNA (11, 27) (**Figure 2B**). CircLigase II is one known enzyme that can attach the ssDNA to another ssDNA before using a primer to convert the molecule into dsDNA. Next, a second adapter ligation is performed using T4 DNA ligase (22). This strategy, although effective, can be expensive and time-consuming (33) since the efficiency of ssDNA ligation to ssDNA is reportedly low (34).

### 3.3 Splinted adapter method

As a follow-up to the ssDNA-LP method for ancient DNA, Gansuage et al. introduced the “ssDNA2.0” (34), which reduced some of the caveats of the earlier RNA Ligase-based ligation (22) by replacing the single-stranded ligation step with a splinted adapter (**Figure 2C**). Here, one side of the double-stranded adapter anneals with the target ssDNA strand through random hybridization with the single-stranded random nucleotide splint (35). This creates a nicked DNA scenario, allowing the use of the T4 DNA ligase instead of relying on the expensive and inefficient CircLigase-based reaction (34).

This method has been adapted to cell-free DNA (19). Similarly, the cell-free DNA application also uses a splinted double-stranded adapter where the bottom strand has a degenerate (or randomer) sequence to hybridize with the single-stranded target on both ends. Single-stranded binding proteins are added to stabilize the ssDNA conformation to facilitate better ligation. Once stably attached, a nick repair ligase (usually T4 DNA ligase) can be used to seal the nick and ligate the adapter. A heat denature step can then be used to remove the bottom strand for downstream reactions. Additionally, the initial adapter ligation can be designed so that it is performed all in one reaction, which reduces the need for multiple clean-up steps.

### 3.4 TDT-assisted adenylate connector-mediated single-stranded DNA ligation

Miura et al. published an improved method for adapter tagging technique using a TdT-assisted adenylate connector to mediate single-stranded ssDNA (TACS) ligation (**Figure 2D**). Similar to TDT-assisted approaches, this technique begins with the initial ribotailing of adenosines (up to 3 bases) at the 3’ end of the ssDNA. However, instead of using the polyA tail for complimentary hybridization, it creatively uses a particular RNA ligase (TS2126 RNA Ligase) (36) to append the desired adapter (37). This strategy is based on the observation that T4 RNA ligase has no preference between using DNA or RNA molecules as the donor molecule during a ligation reaction. In contrast, when considering the acceptor nucleic acid fragment, T4 RNA ligases prefer ligating nucleic acids onto RNA versus DNA (38). Therefore, modifying the ssDNA to become more “RNA-like” appeared to improve efficiency for ligation (39).

Although the TACS method improved upon the concept from the ancient DNA ligase strategy (24), they observed that this method was prone to forming adapter dimers. In situations with low DNA input, these adapter dimers would affect the proportion of useful information acquired from sequencing experiments. Additionally, they realized there were opportunities to improve the efficacy of second adapter ligation. To this end, they elected to forgo T4 DNA ligase and instead used vaccinia virus topoisomerase I (VTopoI) as a ligase enzyme for the second adapter ligation (40). Titling this method TACS-TOPO, they showed that, unlike T4 DNA ligase, VTopoI does not connect the 5’ phosphorylated end of the ssDNA molecule to the 3’ hydroxyl terminal. Instead, it ligates the 3’ phosphorylated end to a 5’ hydroxy end of a target DNA. Therefore, preventing the ligation of an available DNA oligo to a free 5’ phosphorylated end could reduce the occurrence of adapter-dimer formation.

### 3.5 CLAMP-Seq

An alternative atypical method for assessing single-stranded cell-free DNA has been developed, titled circular ligation amplification and sequencing (CLAMP-Seq). In this strategy, the cell-free DNA molecules are first separated by heat denaturation and then circularized (41). Next, using gene-specific primers pre-attached with sequencing adapters, they selectively replicated sequences from genes of interest. The investigators showed that constantly replicating from the original circularized strand reduced the propagation of potential PCR mistakes. This method appears ideal for enriching signals from targeted gene regions if effective primers can be designed to extract signals from all fragment permutations of the genes prior to sequencing.

All in all, the development of various ligation strategies is crucial for the initial step of ssDNA-LP workflow. As techniques and approaches evolve, the efficiency of the ligation step will improve. With these two different library preparation workflows available, researchers can consider which protocol would be suitable for their scientific questions (**Table 1**).

## 4 Observations in cell-free DNA using ssDNA library kit

Several studies have attempted to evaluate differences in cfDNA when processed by ssDNA-LP approach compared to a dsDNA-LP approach for the same DNA extracts (11, 27–29). These initial forays demonstrated that the ssDNA-LP is more inclusive to cfDNA for a broad range of types and lengths (**Figure 1B**). These reports suggested that a considerable fraction of cfDNA is non-nucleosomal in fragment size and could be the result of nuclease degradation (29). Both library preparation techniques showed a similar peak in mncfDNA at a dominant peak at 167bp. A 10.4 bp periodicity was also observed in the ssDNA kit but was offset by a 3bp rightward shift. This was attributed to the non-end repair nature of the ssDNA ligation, which may better showcase the nature of the ends of the original fragments.

### 4.1 The presence of ultrashort single-stranded cell-free DNA in plasma

**Figure 3:**
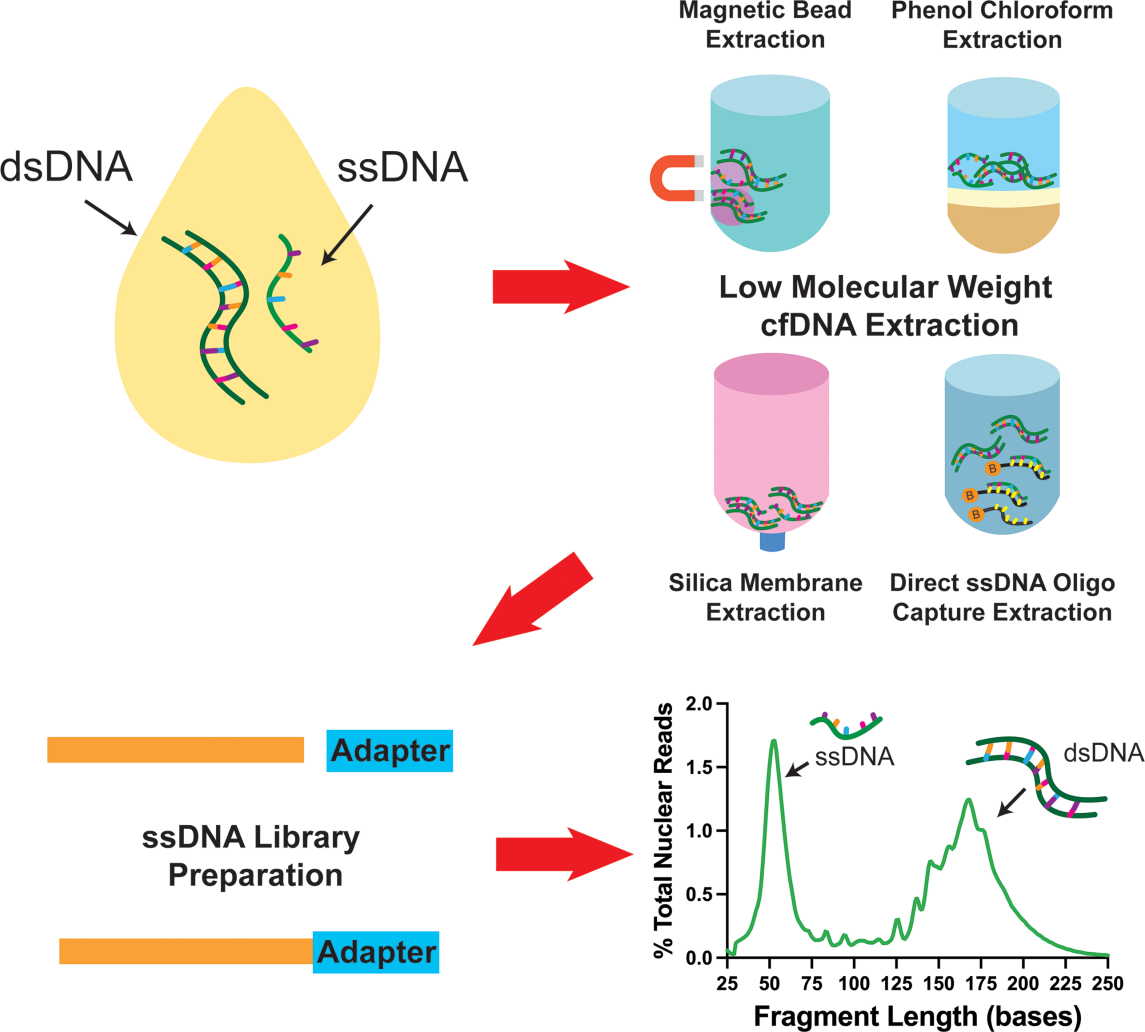
Schematic diagram showing that pairing various low molecular weight enriched extraction methods with ssDNA library preparation reveals the presence of ultrashort single-stranded cell-free DNA in plasma. Data has been derived from (11, 21).

In addition to the major impact that ssDNA-LP had on the presentation of cfDNA, another important aspect is the effect of DNA extraction. Recently, multiple research groups reported the presence of ~50nt ultrashort single-strand DNA (uscfDNA) fragments in both the plasma from non-cancer and cancer patients (21, 37, 42, 43) (**Figure 3**). This novel population of cell-free DNA was revealed by pairing a low-molecular-weight optimized DNA extraction method (**Table 2**) with an ssDNA-LP (**Figure 3**).

**Table 2:** Summary of uscfDNA extraction methodologies.

	Commercial Kit Available	Extraction Principle	Duration	uscfDNA Yield	DNA Populations Recovered when combined with ssDNA-LP
QIAmp Circulating Nucleic Kit (miRNA Protocol) (21)	Yes	Silica Column Based	2 to 4 hours	N/A	Oligonucleosomal cfDNA and uscfDNA
SPRI Extraction Protocol (21)	No	Magnetic Bead and Phenochloroform DNA Precipitation Based	2 hours and requires overnight incubation	N/A	Oligonucleosomal cfDNA and uscfDNA
QIAsymphony DSP Circulating Nucleic Acid Kit (high affinity magnetic bead protocol) (42)	Yes	Magnetic Bead Based	2 to 5 hours	N/A	Oligonucleosomal cfDNA and uscfDNA
Phenolchloroform extraction, and isopropanol precipitation (PPIP method) (37)	No	Phenolchloroform DNA Precipitation Based	1 hour	N/A	Oligonucleosomal cfDNA and uscfDNA
Direct Capture with Degenerate Biotinylated Probes (43)	No	Hybrid-capture	4 hours	N/A	Mainly uscfDNA

### 4.2 Characteristics of uscfDNA

Our group demonstrated that using either the microRNA protocol (referred to as QiaM) of the commonly used Qiagen Circulating Nucleic Acid Kit (44), which uses additional isopropanol and buffer volume, is able to greatly enrich short and single-stranded nucleic acids (characteristics of miRNA) (21). Additionally, we showed that using solid phase reversible immobilization beads (SPRI) with high volumes of isopropanol and crowding agent (polyethylene glycol (PEG)) and salt with phenol-chloroform also promotes the retention of short single-stranded molecules during extraction. Similarly, other investigators showed various methods such as conventional phenol-chloroform-based extraction method (37) or magnetic beads with a commercial nucleic acid extraction kit (42) could also retain these species of cfDNA (**Table 2**). Another unique method reported used 10nt biotinylated capture probes with randomized nucleotide bases to directly capture random single-stranded cell-free DNA in plasma (43).

### 4.3 uscfDNA to mncfDNA ratio quantification challenges

If the size-distribution ratio of uscfDNA and mncfDNA are considered, the phenol-chloroform method (37) apparently recovers uscfDNA at similar efficiencies observed in the QiaM and SPRI extraction methods (21). In contrast, due to this bias toward short single-stranded molecules, the direct hybrid capture method resulted in a very high uscfDNA: mncfDNA ratio (43). This could be explained by the nature of the method, which has a lower affinity for double-stranded mncfDNA. The magnetic bead protocol (42) demonstrated a ratio where the peak of uscfDNA was slightly lower than the mncfDNA. These similar but varied results indicate that although key principals are required to visualize uscfDNA, their representation is still contingent on the method of extraction and library preparation. Therefore, evaluating the efficacy of the extraction between the five methods would be valuable. Currently, there are no methods to quantify uscfDNA from the heterogeneous pool of purified DNA specifically. Commonly used fluorescent-based DNA quantification methods measure total DNA (45), which would not provide any ratio relationships between uscfDNA and mncfDNA. NGS can inform on the ratio between these two species but requires careful spike-in experiments to clarify the recovered concentration compared to the spiked-in amount. In one study, spike-in with oligos of various sizes as a reference suggested that the uscfDNA are present at a concentration of 2.0 ng/ml (43). Total cell-free DNA has been reported to range from 0 to 2000ng/ml (46). Therefore, it is unclear if the concentration of 2.0ng/ml for uscfDNA should be viewed as a minor or major contributor. Hence, at this time, it is difficult to assess the actual uscfDNA concentration without developing new strategies.

### 4.4 Strandedness

**Figure 4:**
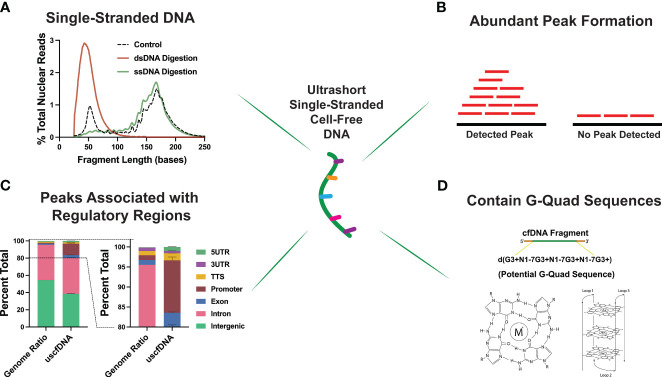
Unique properties of plasma ultrashort single-stranded cell-free DNA (uscfDNA). **(A)** Digestion assays suggest ultrashort cell-free DNA is single-stranded. **(B)** Peak detection bioinformatic tools indicate that uscfDNA maps as abundant peaks along the genome, and these peaks are enriched in **(C)** regulatory regions. **(D)** Sequences of uscfDNA contain potential G-Quad secondary structures. Data has been derived from (11, 21).

Interestingly, through an assortment of deductive experiments, multiple groups inferred that the ~50nt uscfDNA is single-stranded in nature (**Figure 4A**). This was determined by performing strand-specific nuclease digestions on the extracted cfDNA (37) and prior to library preparation (21, 43), revealing that uscfDNA was digestible by ssDNA-specific nucleases (S1 Nuclease and Exo 1 nuclease) but remains intact with dsDNA-specific enzymes (dsDNase) (47). When extracted DNA was processed with the dsDNA-LP, the uscfDNA was not observable, whereas excluding the heat-denature retained the uscfDNA but not double-stranded mncfDNA (21, 37, 42, 43). These experiments provided strong evidence that uscfDNA exists as a single-stranded DNA molecule in circulation.

### 4.5 Genomic characteristics of uscfDNA differ from mncfDNA

As uscfDNA is present in non-cancer individuals, it is physiological but demonstrates distinct characteristics from mncfDNA. Karyograms of the normalized coverage of uscfDNA and mncfDNA populations showed significantly different coverage patterns with uscfDNA mapping to more hotspots within the body of chromosomes and telomeres than the mncfDNA (21, 43, 48). Once aligned, fragments of uscfDNA appear to congregate as peaks in open chromatin regions of the genome, most notably in regions with close proximity to the transcription start sites (TSS), intron, and exonic regions (37, 42, 48) (**Figures 4B,C**). Additionally, compared to mncfDNA, uscfDNA fragments are more colocalized with transcription binding factor sites and histone modification sites (37, 49).

These regulatory regions are also enriched in sequences with a high potential to form secondary structures such as GQuadruplexes (**Figure 4D**). G-Quadruplex structures are observable secondary structures within the chromatin regions of the genome and are correlated to expression levels of oncogenes in the tumor tissue (50, 51). Interestingly, uscfDNA contain a greater abundance of these sequences compared to mncfDNA. Lastly, annotation of the fragment end-motif profiles of uscfDNA reflects the non-random process of nuclease activity (52) and analysis shows that the end-motif profiles are dissimilar between uscfDNA and mncfDNA. Therefore, the properties of uscfDNA (in peak formation in regulatory regions and association with secondary structures) are different from mncfDNA and thus should be considered a separate sub-species of cfDNA. Further exploration could be performed by assessing the animal models of different nuclease knock-down models to observe how they impact the uscfDNA (53, 54).

## 5 Cancer-related differences of ssDNA-LP vs dsDNA-LP

### 5.1 Global fragment size changes in cancer

Early investigations suggested that the fragment profile of tumor-derived cell-free DNA (ctDNA) differs in length compared to those originating from wild-type cells (16, 55). A study showed that cell-free DNA fragments from 90-150bp are enriched in mutation-containing sequences, and by examining these binned sizes in isolation, they can improve ctDNA detection compared to looking at fragments of all lengths (18). The global cfDNA fragment profile can appear aberrated, and analyzing these global changes can be an effective metric for cancer detection (56).

Several studies have performed whole genome sequencing using ssDNA-LP to study the fragment profile of plasma cancer samples (29, 49, 57, 58). They hypothesized that the ssDNA-LP protocol would enrich the diversity of cfDNA molecules and potentially enhance the global fragment differences. To this end, one study compared the dsDNA-LP and ssDNA-LP approach to plasma from lung, breast, liver, and colorectal cancer individuals, showing that the apparent fragment patterns were different than the dsDNA library (although they did not examine paired individuals (58). Here, they established that the ssDNA-LP enriched and revealed a cfDNA fragment population from 30-80bp that was not previously detectable by the double-stranded library. In a follow-up study, they looked at metastatic colorectal cancer to examine if the pattern was able to see differences (29). Different cancer specimens with decreasing amounts of mutant allele fractions (MAF) (68.6%, 54.7%, 47.3%, 23.3%, 14.4%, 3.2%, 0.9%, and healthy) were evaluated, and they observed that both library preparation strategies showed clear differences between non-cancer and cancer subjects. However, their data suggested that ssDNA-LP could show a more pronounced difference. The samples processed with ssDNA-LP had a 10-fold greater number of reads in the small fragment region <100 bases. For example, the highest MAF specimen had a much larger proportion of reads between 30-143bp bases versus the dsDNA-LP, which also showed the trend in fragmentomics but as stated earlier, was apparently less pronounced.

In another study, samples processed with a ssDNA-LP workflow were deeply sequenced at (30-fold of the genome) and also showed a similar fragmentomics difference between colorectal cancer samples compared to non-cancer individuals (49). Therefore, the ability of ssDNA-LP to enrich smaller or single-stranded DNA may provide a better ability for fragmentomic analysis.

### 5.2 Circulating tumor DNA hotspot mutation detection

A report by Liu et al. showed an early attempt to combine ssDNA-LP with hybrid capture to enrich specific mutation-containing fragments in the plasma of 112 pancreatic cancer patients of varying stages (59). Using a custom panel built for 62 pancreatic cancer genes, they found cancer specific mutations in 88% of the samples, and KRAS-specific mutations in 70% of the samples, which was consistent with the tissue-based sequencing. Regarding fragment size, they showed that in pancreatic cancer samples, a substantial proportion of the mutated *KRAS* fragments were shorter than 100 bases. At the same time, the wild type version of those sequences retained their ~167bp modal size. Interestingly, they identified that the decreased footprints were more pronounced in the early stages of pancreatic such as those with intraductal papillary mucinous neoplasm cancer, compared to late stages.

Another paper using Clamp-Seq demonstrated excellent concordance of the detection of hotspot mutations between droplet digital PCR results of 97.4%. Similarly, an analysis of 134 NSCLC patients showed a 94.8% concordance with the tissue genotyping (41).

These studies showed that the ssDNA-LP methods could potentially provide equivalent ctDNA information to the dsDNA-LP methods.

However, one of the caveats of the ssDNA-LP protocol is that it requires the separation of double-stranded molecules prior to adapter ligation. Additionally, natively single-stranded DNA molecules may not have a clear duplex-mate. Therefore, with the current ssDNA-LP workflow, the native duplex information would be inaccessible. Duplex molecule information is often helpful for identifying and removing errors in sequenced reads (17, 60). If only one strand of the duplex reports a variant but not the other, it may be suggestive that the variant arose synthetically during the library preparation, potentially through oxidative DNA damage (61) or cytosine deamination (62). However, other forms of error suppression are still potentially eligible for future development of nonduplex reads. These strategies would likely utilize unique molecular identifier (UMI) correction or bioinformatic in silico error suppression models based on stereotyping experimental data (17).

### 5.3 Copy number variation inference for ctDNA burden

In another body of work, the investigators examined ten samples with high tumor DNA content from eight colorectal patients with high ctDNA % (63). They prepared three kinds of libraries: dsDNA-LP, ssDNA-LP, and pure ssDNA library (no heat denaturation), and the ssDNA-LP was constructed using a TdT-mediated ligation strategy. To evaluate the ctDNA tumor fraction, they developed an algorithm called the plasma genomic abnormality 841 score (PGA) (64). They observed that the ssDNA-LP, pure ssDNA library and ssDNA-LP had greater ctDNA content as per the PGA score. They suggested that the reason for the increased observed ctDNA signal (through PGA) was due to ssDNA-LP’s ability to ligate smaller DNA, pre-existing ssDNA, or nicked DNA. These conformations of DNA were abundant in the plasma of cancer samples, and inclusion could improve cancer signals from plasma.

In contrast, in a letter to the editor, Moser et al. questioned if, when compared to dsDNA-LP, ssDNA-LP could provide a greater ctDNA sensitivity (57). In their pilot study, they applied the ssDNA-LP through the use of RNA ligase-based strategy (27) and assessed the copy number variation signal of the cfDNA from five patients with various cancers (breast, colon, and prostate). However, their experiment failed to detect any significant difference or preferential enrichment in ctDNA.

In conclusion, the assessment of whether ssDNA-LP improves over dsDNA-LP for ctDNA detection is still dynamically ongoing. The preliminary papers are promising, but definitive studies have yet to be carried out. Since large-scale comparative studies have not yet been published, it is still unclear if the ssDNA-LP approach has greater sensitivity or specificity for cancer detection. However, there will likely be attempts to apply the creative approaches for ctDNA detection designed from dsDNA-LP to ssDNA-LP.

### 5.4 Clinical cancer detection potential of uscfDNA

A couple of studies have examined the utility of uscfDNA as a novel biomarker for cancer detection (42, 48). Hudecova et al. explored the properties of uscfDNA between plasma from 21 pan-cancer samples (breast, lung, thymoma, rectal colorectal, and ovarian) and 28 healthy individuals (42), whereas our group investigated alterations in the uscfDNA between 14 late-stage lung cancer and 18 healthy controls.

Regarding changes in the ratio of uscfDNA to mncfDNA changes, despite contrasting directionalities, there appears to be a change in uscfDNA abundance in cancer samples. In samples with higher ctDNA load [using copy number variation as an inference (18)] demonstrated the most observably decrease in uscfDNA abundance compared to other samples (42). In contrast, our group observed an increase in uscfDNA content in late-stage lung cancer samples compared to non-cancer individuals (48). Using copy number variation, Hudecova et al. observed that uscfDNA appeared to contain but was not enriched in the tumor-derived signals (42). Interestingly, both groups found that uscfDNA fragments that promoter regions were enriched in G-quadruplex secondary structure sequences and that this decreased in cancer patients. Additionally, changes in the composition of specific functional element peaks, end-motif profiles, and fragment-size distributions were observed in the uscfDNA population between lung cancer and non-cancer subjects (48). These early studies suggest that the accompanying uscfDNA with the conventional nucleosomal cell-free DNA appears to be a potentially new biomarker for cancer detection.

## 6 Other cfDNA applications of ssDNA-LP

### 6.1 Effect of ssDNA-LP on other biofluids

In the cell-free DNA of other biofluids, such as urine and saliva, single-stranded libraries have also been shown to alter the perceived fragment size characteristics. In cell-free saliva, with similarity to the observations made in plasma, compared to the dsDNA-LP, the ssDNA-LP demonstrated a 3bp rightward shift in the fragment periodicities. Additionally, there was a slightly greater retention of shorter fragments below 100 bases (65). For urine, the ssDNA-LP revealed that the cfDNA was short and fragmented, with a large proportion of fragments below 100 bases. However, in that report, the samples were not directly compared with dsDNA-LP (66).

### 6.2 Effect of ssDNA-LP on non-human species

Using the ssDNA-LP approach, Burnham et al. observed an increase in the proportion of bacteria and mitochondrial (cfmitDNA) content from samples (27). Other groups showed that the low molecular weight DNA extraction also helped enrich the cfmitDNA (21). The enhanced ability to track the profile of bacteria species using cell-free DNA has been effective in monitoring organ transplant outcomes (27, 67).

## 7 Conclusions and future directions

The introduction of robust ssDNA-LP technology has opened new avenues in the realm of liquid biopsy, illustrating the impact  that different methodologies have on the perceived observations. The ability to assess a greater variety of cfDNA species in plasma as well as other biofluids has increased the pool of DNA species to be examined in plasma. Both biomolecular and bioinformatic techniques will need to be developed to harness these new populations of cell-free DNA. More studies will be needed to show if ssDNA-LP pushes the needle of sensitivity compared to dsDNA-LP. Additionally, ultrashort single-stranded cell-free DNA, which appears to have different properties and biological origins compared to mncfDNA, is now added to the toolbox as another potential biomarker for cancer detection. Resultingly, many opportunities are readily available for the development of novel strategies to examine the biological and clinical relevance of the diverse cell-free DNA populations uncovered by the ssDNA-LP approach.

## Author contributions

JC: Conceptualization, Writing – original draft. NS: Conceptualization, Writing – review & editing. DW: Conceptualization, Writing – review & editing. DC: Conceptualization, Writing – review & editing.
